# CenSeam, an International Program on Seamounts within the Census of Marine Life: Achievements and Lessons Learned

**DOI:** 10.1371/journal.pone.0032031

**Published:** 2012-02-01

**Authors:** Karen I. Stocks, Malcolm R. Clark, Ashley A. Rowden, Mireille Consalvey, Thomas A. Schlacher

**Affiliations:** 1 San Diego Supercomputer Center, University of California San Diego, La Jolla, California, United States of America; 2 National Institute of Water & Atmospheric Research, Wellington, New Zealand; 3 Faculty of Science, University of the Sunshine Coast, Maroochydore, Australia; National Oceanic and Atmospheric Administration/National Marine Fisheries Service/Southwest Fisheries Science Center, United States of America

In 2005 the Census of Marine Life launched “A Global Census of Marine Life on Seamounts” (CenSeam), an international science project to increase our knowledge of the ecology of seamounts. Specifically, the mission of CenSeam was to determine the role of seamounts in the biogeography, biodiversity, productivity, and evolution of marine organisms, and to evaluate the effects of human impacts on seamounts. Here we overview the history, goals, activities and programmatic outcomes of CenSeam, with recommendations for improving similar programs in the future. Effective components of the project included mini-grants of generally less than US$10,000 to fund proposal development or difficult-to-fund research, or to expand the scope of an expedition; travel funds for data analysis working groups to meet, several times if needed, to address a targeted research question; advanced training workshops for both young researchers and established scientists; staff support for organizing books, special issues in journals, and review papers; and advising conservation and management initiatives on seamount science. From a programmatic perspective, the lessons learned include the importance of: having the science community develop and endorse the key programmatic and scientific goals; in-person meetings and workshops to foster new collaborations; promoting open data sharing; funding salary time for critical work; and establishing and actively managing open communication mechanisms to allow scientists to develop a consensus opinion on science topics, which could then be conveyed to conservation and management organizations.

## Introduction

Seamounts (undersea mountains) continue to be focal areas for marine science, encompassing research that ranges from plate tectonics, oceanic convective heat budgets, the physical structure and dynamics of the ocean's water masses, and the composition of the ancient atmosphere [1]–[5]. Research into the ecological function of seamounts is equally varied. Several groups of organisms have demonstrated hotspots of elevated biomass over seamounts, including mobile pelagic fauna [6]–[9] and larger invertebrates on the seafloor [10], [11]. Seamounts can act as refugia: as presumably isolated habitats, they create conditions that favour the existence of ‘living fossils’ and, in a few isolated cases, support archaic assemblages that are more similar to fossil strata than extant communities [12]–[14]. This refugia function of seamounts may gain new importance as future, shallow-water refuge areas for deep-water corals that become displaced from deeper layers by changing ocean chemistry [15], [16] (but see [16]). Conventional wisdom previously held that seamounts mimic islands whose biological communities contain more species of small geographic ranges (i.e. ‘endemics’) than other areas of the oceans, though this notion has been challenged in recent studies, including those employing genetic techniques [12], [17]–[19]. Instead, seamount communities, though they have structural differences, may play a dynamic role in the source-sink dynamics of abutting systems [20].

There is widespread consensus that biological components of seamounts are highly vulnerable and sensitive to human disturbance and exploitation [21], [22]. The best documented, most widespread, and presumably most substantial human impacts on seamounts are caused by fishing. The history of fishing on many seamounts and for many seamount-associated fish stocks shows a classic ‘boom and bust’ pattern, with few seamount fisheries appearing to be sustainable in the longer term [23]. The impacts of fishing extend from detrimental effects on fish stocks to the seafloor: benthic communities are frequently composed of long-lived and fragile invertebrates (e.g. corals) that have very low tolerances to physical encounters with fishing gear [24], [25]. Consequently, impacts from bottom-contact fishing can be massive, and recovery times may be in the range of decades to centuries [26]. Mining for mineral deposits on seamounts presents a new, and potentially large, threat to seamount ecosystems [27], and emphasizes a need for global, scientifically robust conservation and management planning for seamounts [28], [29]. The increasing biological research on seamounts [30], coupled with these growing management concerns, led to the founding of the Global Census of Marine Life on Seamounts (CenSeam) in 2005 as part of the Census of Marine Life program.

## CenSeam - a global science programme on seamounts

The Census of Marine Life (CoML) was a 10-year international effort to assess the diversity, distribution, and abundance of marine life on a global scale [31]. Recognizing a global interest in seamount science, the Census of Marine Life funded a workshop in 2003 to evaluate the potential importance and impact of a global project on seamounts under the CoML mantle. This workshop brought together 33 participants from 11 countries representing the research community, national and international resource management agencies, and conservation organizations. The workshop participants concluded that seamounts are an ecologically important yet relatively unexplored habitat warranting further study, and that a devoted CoML field program could have a valuable role in energizing and coordinating seamount research [32].

### 1. CenSeam Science Objectives and Goals

Workshop participants defined a set of research priorities for CenSeam [32]. From the core research priorities identified at the initial workshop, two overarching themes evolved. Within each theme a suite of more specific and tractable questions were identified, as detailed below.

Theme 1: What factors drive community composition and diversity on seamounts, including any differences between seamounts and other habitat types?

- Do community composition and diversity differ between seamounts in different regions, and what environmental factors cause large-scale geographic patterns?
- How important are differences in dispersal capabilities in producing spatial differences in species composition on seamounts?
- What environmental factors (e.g. hydrodynamic regimes, substrate age and type) cause differences in diversity and species composition of seamount fauna at the scale of individual seamounts?
- Are seamounts centers of high biological productivity?
- Are seamounts characterized by unique trophic architecture?

Theme 2: What are the impacts of human activities on seamount community structure and function?

- How vulnerable are seamounts to bottom fishing?
- What are the threats posed by non-trawl (e.g. longlining) fishing activities?
- What are the effects of mining on seamount communities?
- How resilient are seamount communities to human-induced disturbance?
- Are seamounts different from other habitats in their capacity to recover from human-induced disturbance?

CenSeam addressed these research themes by defining 4 key programmatic goals:

1. to coordinate and expand existing and planned seamount research;
2. to foster new field expeditions;
3. to improve data management and data analysis; and
4. to facilitate public education and outreach.

### 2. CenSeam Organization

A Secretariat, hosted at the National Institute of Water & Atmospheric Research (NIWA) in New Zealand, was responsible for overseeing all aspects of the project. The Secretariat comprised three Principal Investigators and a Project Coordinator (Table 1). The CenSeam Project Coordinator was responsible for the day-to-day running of the CenSeam Secretariat, and acted as the project's central point of communication. The Project Coordinator was near full-time, and the remainder of the Secretariat was supported for a minority of their time on the project. A Steering Committee guided the overall direction of CenSeam. This committee consisted of 13 scientists from 11 countries representing a range of expertise, including ecology, taxonomy, genetics, geology, and oceanography (Table 1). Two smaller groups, the Data Analysis Working Group (DAWG), and the Standardization Working Group (SWG), were also formed early in the project. The DAWG identified gaps in the current knowledge of seamounts, and undertook and provided guidance for the analysis of existing data. The SWG developed recommendations for standard sampling and data reporting practices. Both these groups had a core membership which was augmented as required for particular tasks.

**Table 1:** CenSeam Committees and Membership.

**CenSeam Secretariat**
Malcolm Clark, New Zealand (Principle Investigator); Mireille Consalvey, New Zealand (Project Coordinator); Ashley Rowden, New Zealand (Principle Investigator); Karen Stocks, United States (Principle Investigator)
**CenSeam Steering Committee**
Amy Baco-Taylor, United States; John Dower, Canada; Baban Ingole, India; Tony Koslow, Australia (former); Gui Menezes, Portugal; Tina Molodtsova, Russia; Bertrand Richer de Forges, New Caledonia; Alex Rogers, United Kingdom; Thomas Schlacher, Australia; Timothy Shank, United States; Shinji Tsuchida, Japan; Martin White, Ireland; Alan Williams, Australia; Ian Wright, United Kingdom;
**CenSeam Data Analysis Working Group (DAWG)**
Amy Baco-Taylor, United States; Paul Brewin, United States (former), Malcolm Clark, New Zealand; Timothy O'Hara, Australia; Ashley Rowden, New Zealand (DAWG Facilitator); Alex Rogers, United Kingdom; Thomas Schlacher, Australia; Karen Stocks, United States; Derek Tittensor, Canada
**CenSeam Standardisation Working Group (SWG)**
Malcolm Clark, New Zealand (SWG Facilitator); Mireille Consalvey, New Zealand (SWG Facilitator); John Dower, Canada; Gui Menezes, Portugal; Bertrand Richer de Forges, New Caledonia; Alex Rogers, United Kingdom; Thomas Schlacher, Australia; Timothy Shank, United States; Alan Williams, Australia

### 3. CenSeam Activities and Outcomes

The primary activities and outputs of CenSeam (Tables 2, 3, 4, 5) are described below. We provide opinions about the efficacy of each component from the perspective of the Secretariat, but recognize that these evaluations are to some degree subjective. Nevertheless, they are offered with the intent that they may inform future projects. Larger programmatic conclusions are given in the final section on lessons learned.

**Table 2:** Primary CenSeam research publications.

Books
Seamounts: Ecology, Fisheries and Conservation [33]
Biological Sampling in the Deep Sea, in preparation for Wiley Blackwell by M. R. Clark and M. Consalvey
Reviews and Book Chapters
The ecology of seamounts: structure, function and human impacts [22]
Life on Seamounts [43]
Special Issues
Mountains in the Sea, Oceanography 23(1) 2010. CenSeam collaborated with the Seamount Biogeociences Network to produce this issue
Recent Advances in Seamount Ecology, Marine Ecology 31(S1) 2010
The CenSeam Collection, PLoS ONE 2011
Data Analysis Papers*
Deep-sea coral collection protocols [46]
Seamounts, deep-sea corals and fisheries: vulnerability of deep-sea corals to fishing on seamounts beyond areas of national jurisdiction [47]
Historical deep-sea coral distribution on seamount, oceanic island and continental shelf-slope habitats in the NE Atlantic [48]
Cold-water coral habitats on seamounts: Do they have a specialist fauna? [41]
Are deep-sea demersal fish assemblages globally homogenous? Insights from seamounts [49]
Assemblage structure, but not diversity or density, change with depth on a northeast Pacific seamount [50]
Conflicting estimates of connectivity among deep-sea coral populations [51]
Environmental drivers of ophiuroid species richness on seamounts [52]
Paradigms in seamount ecology: fact, fiction and future [18]
Seamount megabenthic assemblages fail to recover from trawling impacts [26]
The global distribution of seamounts based on 30 arc seconds bathymetry data [42]
Predicting global habitat suitability for stony corals on seamounts [44]
A test of the seamount oasis hypothesis: seamounts support higher epibenthic megafaunal biomass than adjacent slopes [53]
Squat lobster assemblages on seamounts differ from some, but not all, deep-sea habitats of comparable depth [54]
Effect of deepwater trawling on the macro-invertebrate assemblages of seamounts on the Chatham Rise, New Zealand [55]
Impacts of bottom trawling on deep-coral ecosystems of seamounts are long-lasting disturbance [24]
An index to assess the risk to stony corals from bottom trawling on seamounts [28]
Incongruent patterns of genetic connectivity among four ophiuroid species with differing coral host specificity on North Atlantic seamounts [56]
A global seamount classification to aid the scientific design of marine protected area networks [45]

*These publications were either supported by CenSeam minigrants, came out of Data Analysis Working Group activities, or acknowledged CenSeam inputs.

**Table 3:** CenSeam conservation and management activities and outputs (in addition to research publications).

*Consultation and Advising*
Participation in the FAO Expert Consultation on Management of High Seas Fisheries
Advising the developing South Pacific Regional Fisheries Management Organization (SPRFMO), including establishing detection criteria for managing trawl impacts on vulnerable marine ecosystems [57]
Participation in an International Seabed Authority (ISA) workshop that gathered data and identified knowledge gaps for the diversity and distribution patterns of seamount fauna on cobalt-rich crusts, and several additional ISA meetings.
Participated in or presented at the International Marine Conservation Congress (IMCC), 5th World Fisheries Congress, and meetings of the United Nations Environment Programme (UNEP), and International Union for Conservation of Nature (IUCN) (2008 and 2009)
Feedback to the IUCN on the application of scientific criteria adopted by parties to the Convention on Biological Diversity (CBD) to identify Ecologically and Biologically Significant Areas (EBSAs) of the open ocean and deep sea in need of protection, and participation in the development of illustrations for CBD criteria and methods for the identification of seamount EBSAs [58].
*Reports*
Vulnerability of deep-sea corals to fishing on seamounts in areas beyond national jurisdiction [47], a report to the United Nations Environment Programme
Assessment of the Conservation Values of the Norfolk Seamounts Area: a component of the Commonwealth Marine Conservation Assessment Program 2002–2004 [59], a report to the Australian Government Department of the Environment and Heritage
The biodiversity of cobalt-rich ferromanganese crusts [60], [61], reports to the International Seabed Authority
International Guidelines for the management of deep-sea fisheries in the high seas [62], a report for the IUCN to the FAO
Contributions to the first global oceans and deep seabed biogeographic classification [63], a UNESCO report
Connectivity and conservation of Australian and New Zealand seamounts: a molecular approach to assess relationships among their deep sea coral populations [64], a report to the Australian Department of Environment and Heritage

**Table 4:** Primary CenSeam education and outreach activities.

*Conferences Special Sessions*
CenSeam researchers initiated and chaired 12 special sessions at scientific conferences. Dedicated seamount sessions at the European Marine Biology Symposium (EMBS) marked the start (2005) and end (2010) of CenSeam.
*Workshops*
CenSeam Image Analysis Workshop. May 2007, Totnes, UK
Classification and Identification of Marine Organisms from Images and Video. March 2009, Monterey Bay Aquarium Research Institute, USA
*Education and Outreach*
CenSeam Newsletters
Archive of images and video for use by educators and the media (http://censeam.niwa.co.nz/node/371)
Voyage Logs (http://censeam.niwa.co.nz/voyages)
Press coverage for science results, e.g. “City of Brittlestars” (http://www.coml.org/comlfiles/press/CoML_CenSeam_Public_Release-05-16-08.pdf)

**Table 5:** CenSeam by the numbers: outputs of CenSeam and CenSeam-affiliated researchers.

8	flagship cruises
12	special sessions organized or co-organized at conferences
15	theses and dissertations produced by CenSeam-affiliated scholars
16	workshops organized or co-organized
30	countries with participants
59	taxonomists in CenSeam Taxonomy Network
60	invited seminars, speeches and briefings
∼100	active members (those on working groups, authoring or editing articles or issues, receiving mini-grant funding, participating in analysis project, etc.)
122	expeditions joined by CenSeam-affiliated researchers
228	publications by CenSeam-affiliated researchers
∼500	people receiving the CenSeam newsletters
∼$20,000,000	in funding to CenSeam-affiliates for seamount-related research

#### 3.1 Networking

One of CenSeam's overarching aims was to create a global network of seamount researchers. Engagement and communication was critical to the success of this aim. CenSeam newsletters and shorter emailed news items provided updates on CenSeam activities, new scientific findings, seamount management and conservation happenings, upcoming conferences, announcements of opportunities such as postdoctoral positions and open berths on research voyages, profiles of seamount community members, and features on newly described or interesting seamount organisms. About 500 people registered to receive the CenSeam newsletter, indicating a substantial level of interest in this mechanism for sharing community news.

CenSeam developed a network of taxonomists, covering a wide range of taxa and regions, who were willing to process and identify specimens from seamounts using both traditional and genetic approaches. Field programs were invited to use the list to supplement their available taxonomic expertise, and thus ensure more complete analysis of the taxonomic groups within their samples. Whilst taxonomists showed a willingness to be a part of the network, it was not widely utilized, perhaps indicative of research institutes already having in-house expertise, or having established their own networks of expertise.

#### 3.2 Mini-Grants

CenSeam provided small grants (generally <US$10,000) to fund activities that furthered CenSeam's goals and science objectives. The mini-grant proposal process was designed to minimize the workload on both sides: the proposals were short, the funding rate relatively high (almost 60%), and an additional pool of discretionary funds was available for time-sensitive projects. Proposals submitted in response to the annual solicitations were reviewed and voted upon by Steering Committee members; discretionary fund requests were reviewed by the secretariat.

Mini-grant projects included digitization and quality control of previously-inaccessible historic data, travel for scientists to join otherwise-funded expeditions, salary or travel for taxonomists to identify previously unidentified specimens from seamounts or to standardize identifications across expeditions, and salary or travel to augment data analyses.

In the opinion of the Secretariat, the mini-grants had a high impact per dollar and were a cost-effective mechanism for furthering the goals of CenSeam. The mini-grant program targeted work that was difficult to fund under traditional funding schemes, either because it was too small for a major proposal effort, required too rapid a decision making process, or had a scientific focus that did not match national science funding priorities. The mini-grants were also highly accessible to early-career researchers, helping to further the next generation of seamount scientists. Examples of the scientific outputs of the minigrants can be found this special CenSeam collection in PloS ONE, a special issue of Marine Ecology [1] and Table 2.

#### 3.3 Stand-Alone Conferences and Special Sessions at Conferences

As well as presenting CenSeam results at numerous international conferences, CenSeam researchers instigated, planned and represented the project at 12 special conference sessions; most notably at the European Marine Biology Symposium in 2005 and 2010, to mark the start and end of the CenSeam programme. Special sessions proved a valuable opportunity to showcase CenSeam research, focus research attention on particular scientific questions, and increase awareness of and participation in CenSeam activities. CenSeam also sponsored a planning meeting for the book *Seamounts: ecology, fisheries and conservation* [33], bringing together a wide cross-section of the seamount community to facilitate the writing of the book. This working meeting, extending over several days, proved valuable in establishing cross-disciplinary linkages and having in-depth discussions of the current state of seamount science. While formal conferences gave opportunities to present recent research, the longer and more informal working meetings were more effective at fostering new research collaborations.

#### 3.4. SeamountsOnline, a global database of seamount biology

The goal of SeamountsOnline [34] was to bring species occurrence data from multiple seamounts and studies into one integrated web-accessible database to support scientific analyses and management decisions. From the first year of CenSeam, SeamountsOnline was serving data online. Throughout the tenure of CenSeam, SeamountsOnline's data holdings tripled and new features were added to the portal, such as a map interface for querying and accessing data. Its database structure became a model for other Census of Marine Life programs setting up databases, as well as for the SYNDEEP, the CoML's cross-habitat deep sea biodiversity study.

SeamountsOnline supported some of the early gap analysis work that informed CenSeam priorities, such as creating the first global map of biological sampling on seamounts. Data from SeamountsOnline were also used for analyses [35]–[37], planning future expeditions [38], [39], and to support the data evaluation stages of some of the DAWG analyses. Despite best efforts, SeamountsOnline only holds a fraction of the world's seamount data. The completeness of SeamountsOnline was limited by the person support needed for time-consuming data entry (particularly digitization of older, hard-copy reports). A further challenge was the lack of a well-established culture of data sharing in the deep-sea biological community, in contrast to, for example, astronomy, genetics and physical oceanography. Nor are there widely-adopted standards and formats for data and data description. While researchers were supportive of the concept in many cases, limits to data sharing included the understandable prioritization of funded project deliverables over the generally-unfunded work of preparing datasets for sharing, a lack of clear community agreements for crediting data providers (some CenSeam scientists expected co-authorship in any paper using their data, but were offered only citation and acknowledgement), a desire to retain ownership of data until all publications were produced (a process that could span many years), and a perception that SeamountsOnline was not complete enough or flexible enough to meet real analysis needs, particularly in its earlier years.

Data-sharing was improved when CenSeam directly funded data contributions through mini-grants. Researchers were also more likely to contribute data when a specific analysis goal was identified, and the data providers were actively involved in the analysis and any forthcoming publications.

#### 3.5 Data Analysis Projects

CenSeam decided early on to actively facilitate meta-analyses across multiple and often disparate datasets. These challenging analysis projects were undertaken under the auspices of the DAWG, fueled by CenSeam funds that allowed small groups to meet at intervals for 2–3 day workshops. Groups were generally less than 10 people, selected for their access to required data, or their biological, statistical, or modeling expertise.

A substantial number and diversity of analysis projects were undertaken and completed within the lifetime of CenSeam (Table 2), and the scientific output as measured by publications was high compared to the costs. These projects generally focused on filling critical gaps in the scientific knowledge of seamounts, providing targeted conservation or management advice, or taking advantage of valuable available data or expertise. The DAWG organization structure is considered instrumental in the success of the data analysis projects. Though most DAWG members were not funded beyond travel expenses, they received professional recognition for publications coming out of DAWG. In addition, participants found value in the DAWG meetings as opportunities to discuss their individual research projects with colleagues, and consider ways to enhance their planned analyses, which fostered additional projects not explicitly supported by CenSeam [40]–[42]. CenSeam generally selected somewhat isolated and interesting locations to encourage focus and camaraderie within the group, and provide a small additional incentive for involvement. However, there was also a cost to this approach: the additional travel time for a somewhat more remote location occasionally made participation difficult for those with restricted schedules.

#### 3.6 Training Workshops

Photos and videos have become widely used tools to complement, or replace, physical collections of organisms, especially in deep waters. However, there is often large variability in image collection techniques, gear types, survey designs, storage, documentation, and processing methods. In 2007, CenSeam organized and funded a workshop to bring together 24 international representatives to further the standardization of practices through a detailed discussion of image acquisition, survey design, data management, and image analysis. The meeting provided an opportunity for researchers to share knowledge that was much more technical and detailed than normally published in the primary literature or presented at a scientific conference.

One outcome of the 2007 workshop was a recognition of the wide need for improved training on the identification of organisms from video. To meet this need, CenSeam partnered with the Monterey Bay Aquarium Research Institute (MBARI) in 2009 to offer a 3-day training workshop. Taxonomic experts covering most of the major deep-sea faunal groups trained 65 participants, including one-on-one sessions for participants bringing their own photos or video. The workshop facilitated the exchange of information and ideas between institutes, trained early-career researchers, and ultimately enabled a greater level of confidence when assigning taxonomic identifications to deep-sea fauna seen in images. The collaborative nature of this workshop was particularly successful: MBARI provided the expertise and location; CenSeam provided funding and logistical support, as well as the infrastructure to advertise it to the international seamount community; and the many participating taxonomists freely shared their knowledge. To further distribute the knowledge, information from the workshop is being included in a chapter on the use of towed cameras, led by David Bowden, in the forthcoming book Biological Sampling in the Deep Sea from Blackwell Publishing. The interest in these particular workshops may indicate a larger need for specialized training opportunities in advanced techniques, both for young scientists and career professionals.

#### 3.7 Seamount Voyages

In 2006 the first CenSeam-linked voyages sailed to seamounts off New Zealand, initiating the field research component of CenSeam. CenSeam researchers from many nations have conducted field work across several regions (Atlantic, Pacific, Mediterranean, Southern Ocean), taxa (from corals to cetaceans), and spatial scales (from individual seamounts to basin-scale comparisons), employing a wide range of technologies, including satellite imagery and ROVs. Many of the voyages have been multi-disciplinary, epitomized by a 2008 “flagship” voyage to the Macquarie Ridge (Southern Ocean) that brought together an international team of biologists, physicists and geologists.

The scale of CenSeam funding did not allow core funding for research expeditions; instead, CenSeam focused on influencing and expanding the scope of global seamount sampling. At its inception, CenSeam compiled the first global map of seamount sampling and highlighted that the Indian Ocean, South Atlantic, and the Western and Southern Central Pacific were the most under-sampled regions and hence priorities for new expeditions. CenSeam support contributed to securing funding for expeditions to two of these regions—the Indian Ocean and the South Atlantic—as well as other regions. CenSeam mini-grants have also added value to seamount voyages by providing travel funding to fill open berths on expeditions, and equipment loans to expand the scope of sampling.

While the 5 year timespan of CenSeam is long in relation to many science projects, it was not sufficient to allow the results from expeditions catalyzed by CenSeam to yield their results and guide further CenSeam research; the analysis and expedition components operated largely in parallel. A longer program duration would have allowed an iterative process of DAWG analyses feeding into new expedition ideas, and expedition results shaping new analysis projects.

#### 3.8 Public Education and Outreach

**Figure 1:**
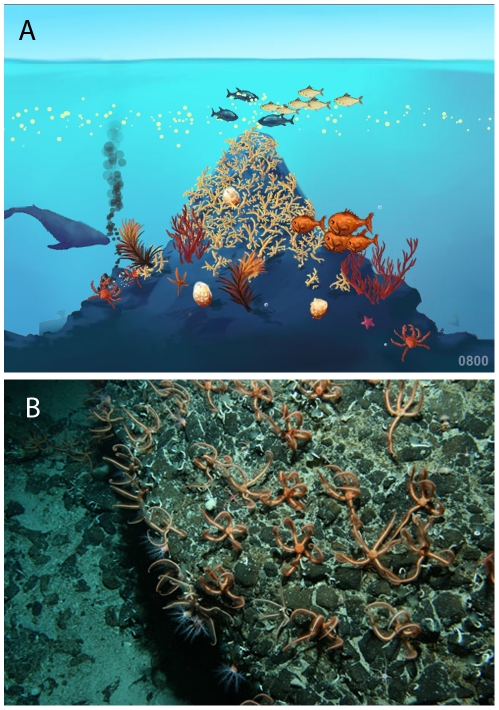
Examples of seamount images produced by CenSeam for use by educators and the media. A) Diagram of a seamount community, showing the primary components and zonation; B) Photo from the “City of Brittlestars” expedition. Credit: CenSeam-NIWA.

CenSeam efforts to promote deep-sea science to the general public took several forms. As part of the Deep Sea Education and Outreach (DESEO) initiative, CenSeam collaborated on the expansion of a touring museum display, *Deeper than Light*, which has received more than one and a half million visitors. Press releases alerted the media to interesting or important findings from CenSeam researchers. A prime example was the discovery of a “city of brittlestars” atop a seamount on the Macquarie Ridge. This press release attracted considerable media attention with reports in 26 countries and 7 languages in newspapers, television, radio, and online media. At a local level, CenSeam researchers gave presentations to school and university groups, as well as interested public groups. The CenSeam secretariat made high quality images available for use by educators and the media (Fig. 1). Eight cruises during the tenure of CenSeam provided regular “ship to shore” voyage logs that were posted on the CenSeam website (http://censeam.niwa.co.nz/), and received tens of thousands of hits, and were featured in school curricula. The overall Census of Marine Life website and education and outreach effort provided additional exposure for CenSeam news.

Overall, the CenSeam outreach effort was limited by the lack of experience many researchers have in working with the media and public, and the low professional rewards academic institutions give to outreach in comparison to research outputs. The design and maintenance of the website also proved to be a larger task than originally planned. The most successful components, such as the museum display and the City of Brittlestars press release, were done in collaboration with education or outreach professionals.

#### 3.9 Books, Special Issues of Journals Review Papers

CenSeam actively worked to create compilations, syntheses and summaries of the current state of seamount science for various audiences, in addition to fostering individual original research publications. These included a review in Annual Reviews of Marine Science [1], a chapter on seamounts in the Census of Marine Life book [43], and special issues published by the journals Oceanography and Marine Ecology (references given in Table 2). The CenSeam special collection in PLoS ONE, the latest to date, incorporates a number of research papers produced during the last months of CenSeam. While many seamount papers would have been published without CenSeam, the resources CenSeam provided to support salary and travel costs for in-person planning, proved important for organizing larger projects such as books, major reviews, and special issues.

#### 3.10 Contributions to Global Conservation and Management Initiatives

Throughout the project, CenSeam members carried out and interpreted science to inform the management of commercial fisheries and mining on seamounts, as well as the conservation of seamounts. Data and advice were delivered via a spectrum of meetings, workshops and reports that addressed the specific needs of national and international management organizations (Table 3). In addition to communicating existing science to conservation and management efforts, CenSeam also used mini-grant funding to support new analyses and to fill critical gaps in understanding and new tools to support management (e.g. [40], [44], [45]). Though advising conservation and management was not one of the original goals of CenSeam, it became an area of high activity and demand. This can be attributed in part to the connections that a few key CenSeam researchers already had with management and conservation agencies, and in part to the usefulness of having a central point of contact for sound scientific advice and data.

### 4. Summary of Outcomes

At the outset of CenSeam, our understanding of seamount ecosystems was limited by significant gaps in geographic coverage of seamount sampling, varied approaches to data acquisitions, unstandardized sampling methods, and a lack of large-scale syntheses. We believe that CenSeam has been instrumental in connecting, focusing, and collating the efforts of many international researchers. It has facilitated a wide scope of activities, ranging from field sampling to data mining and numerical analyses and syntheses.

Some of CenSeam's key outcomes include having:

- Initiated and strengthened global collaborative research;
- Expanded global seamount sampling to regions with little biological sampling;
- Provided high-quality science to inform conservation planning and resource management of seamounts;
- Developed the first global, integrated database of seamount biogeography;
- Enhanced discovery and taxonomic description of new species;
- Enhanced training, especially in the area of species identification;
- Increased scientific and public awareness of seamounts and the wider deep-sea environment;
- Shaped a new set of paradigms about seamount ecosystems;
- Supported scientific research and publications, which together shaped a new set of paradigms about seamount ecosystems.

### 5. The Future of CenSeam

CenSeam's funding under the Census of Marine Life program ended in 2010. Without it, many of the core activities of CenSeam will not continue. However, it is recognized that the international network of collaborations developed within CenSeam, particularly the working groups, is an important resource that should not be allowed to dissipate. Many of the individual collaborations between researchers, as well as national-level research programmes, will continue to capitalize on linkages developed through CenSeam. In addition, CenSeam researchers have joined with scientists from other deep-sea projects of the Census of Marine Life to launch INDEEP, the International Network for Scientific Investigations of Deep-Sea Ecosystems. This new effort represents a logical progression from CoML's habitat-focused projects: now that each CoML field project has worked to understand an individual oceanic realm, INDEEP will develop and synthesize our understanding of deep-sea global biodiversity and function, and provide a conduit to inform sustainable management strategies.

## Beyond Seamounts: Lessons Learned for Large-scale Collaborative Science

With the end of CenSeam, it is relevant to ask not just how CenSeam's scientific findings can inform future seamount science [1], [4], but also how it can inform the organization of future science programs of similar scope and conceptual goals. Some of the key lessons learned by the CenSeam leadership are described below.

### Establish program goals based on community input

The central scientific and programmatic goals of CenSeam were established by a community workshop. By allowing the community to define the work that it felt was most important and interesting, it garnered enthusiasm for participation despite the limited funding CenSeam could provide. The structure of the CenSeam program and the secretariat were important for organizing and facilitating progress, but the guiding scientific direction came from the scientists themselves.

### Create and maintain collaborations with in-person contact

One of the strengths of CenSeam was its ability to create new collaborations among researchers. In-person meetings of the working groups, and at workshops and conferences, led to discussions and cooperation between researchers that would not otherwise have met. For example, the DAWG brought together modeling, statistics, data management, taxonomy, ecology, and fisheries expertise to address highly complex scientific questions. Though travel costs were quite high, we considered in-person contact necessary to launching and maintaining CenSeam activities. Funds for this sort of travel are often limited under projects funded by traditional scientific research organizations.

### Fund salaries for critical expertise

Most CenSeam activities were voluntary, without salary support for participants. However, two of the successful aspects of CenSeam—data analyses and SeamountsOnline—received financial support. A PhD/postdoctoral researcher with statistical and modeling expertise was supported to participate in key DAWG activities (a related CoML program, FMAP, provided the funding). SeamountsOnline was led by an informatics researcher with partial support for a data manager. Important outputs of CenSeam can be attributed to this funding. However, collaborations with, for example, physical oceanography and marine geological communities, never formed to the same degree, and perhaps would have benefitted from targeted funding to promote collaborative analyses or other a activities. In many cases, supportive institutions allowed the CenSeam secretariat and involved members to devote substantially more time to CenSeam activities than was funded, but additional salary support would have allowed greater engagement.

### Promote data sharing and long-term preservation

SeamountsOnline created and made available the first and largest global collection of seamount data, fed in part by mini-grants funding the rescue of select high-value datasets that would not otherwise be available to the scientific community. However, the original goal of having wide sharing of seamount data was never reached. Very few seamount researchers not directly supported by CenSeam shared their data, even after publication. The impediments to data access (see section on SeamountsOnline, above) are limiting the progress of seamount ecology, and deep-sea biology in general. This is particularly true as the field moves from describing patterns and processes on a local scale, which can be studied by a single expedition, to evaluating commonalities and differences across regional and global scales, which requires the integration of many studies.

Considering the enormous expense of launching new deep-sea expeditions, the loss of the majority of data after the initial publication of results represents a major inefficiency. As a largely voluntary organization, CenSeam had limited ability to provide incentives for data sharing beyond mini-grants; programs funding the collection of data might more effectively create requirements for contributing data to publicly-accessible repositories.

### Creating and expressing consensus in a research community can powerfully influence science and management

CenSeam proved effective at identifying and methodically addressing research themes on spatial scales that exceeded any national project's. This led to a re-evaluation of some of the previously dominant seamount ecology paradigms [18]. Similarly, by developing community-agreed research priorities, CenSeam gave researchers compelling, citable justifications for new field research proposals, influencing the direction of national-scale research priorities.

One of the most successful outcomes of CenSeam was advice to management bodies (Table 3). We attribute CenSeam's success in this area to a combination of the need within the management community–seamount issues became important on several national and international fronts during the tenure of CenSeam–and the value of having a credible, respected, centralized contact point. Organizations were able to ask CenSeam to provide a general scientific consensus on a topic, when one existed, or references to an appropriate expert. CenSeam decided early on to provide scientific input into decision making, but not to lobby for particular management actions or approaches, which allowed it to be recognized as an objective source by conservation and government organizations. The ability to provide a community consensus for seamount scientists was highly valued by conservation and management, as indicated by the number of requests for input, but was labor intensive: creating a consensus position required that all participants have the opportunity to express their views, and multiple iterations were often required before discussions reached a point of agreement.
